# Challenges and strategies to enhance participation in the Iranian medical students’ scientific Olympiad: a qualitative study

**DOI:** 10.1186/s13104-026-07751-4

**Published:** 2026-02-28

**Authors:** Mahdi Abbasi, Seyed Jamal Mirmousavi, Mehdi Rabiei, Sana Jafarlou, Hamid Sadeghi, Elham Navipour

**Affiliations:** 1 Noncommunicable Diseases Research Center, Sabzevar University of Medical Sciences, Sabzevar, Iran; 2 Elderly Health Research Center, Sabzevar University of Medical Sciences, Sabzevar, Iran; 3 Student Research Committee, Sabzevar University of Medical Sciences, Sabzevar, Iran; 4 Isfahan University of Medical Sciences, Isfahan, Iran; 5 Shiraz University of Medical Sciences, Shiraz, Iran

**Keywords:** Scientific Olympiad, Medical students, Engagement, Facilitators, Participation

## Abstract

**Objective:**

This study aimed to explore the challenges and identify strategies to enhance participation in the Iranian Scientific Olympiad for Medical Students. This qualitative descriptive study was conducted using semi-structured interviews with 28 participants, including students, faculty members, administrators, and Olympiad officials. Participants were purposively sampled, and data were analyzed using conventional content analysis. Trustworthiness was ensured through reflexive practices and triangulation.

**Results:**

Student participation was influenced by interconnected individual, structural, and process-related factors. Individual barriers included low self-confidence, competing academic and financial priorities, and limited intrinsic motivation. Structural challenges involved inequitable access to educational resources, insufficient institutional support, limited stakeholder involvement, and perceived lack of transparency in evaluation processes. Process-related barriers included frequent procedural changes, inadequate preparation opportunities, limited mentorship, and unclear feedback mechanisms. Suggested strategies included strengthening mentorship systems, improving transparency and fairness in assessment, increasing stakeholder involvement in decision-making, establishing sustainable institutional support, and developing a centralized and permanent information platform to enhance awareness and engagement.

**Supplementary Information:**

The online version contains supplementary material available at 10.1186/s13104-026-07751-4.

## Introduction

### Background

Human resources are central to health system performance, as inadequate workforce performance can compromise care quality and patient satisfaction [1]. Performance is shaped by factors such as ability, knowledge, and skills, and their effective management can improve health system outcomes [2, 3]. In this context, talent refers to high-level performance developed through systematic learning, and talent management involves identifying and developing individuals with high potential to enhance institutional performance [4, 5]. In medical education, Olympiads and Science Olympiads are structured academic competitions that promote scientific knowledge, problem-solving, and scholarly engagement through individual and team-based challenges [6].

Science Olympiads represent a key platform for identifying and developing talented students. Beyond fostering scientific competition, these events provide opportunities for individual growth, teamwork enhancement, and scholarly exchange among participants [7]. The Iranian Scientific Olympiad for Medical Students is a national academic program designed to identify and support talented students across medical and allied health disciplines through individual and team-based scientific competitions. A brief overview of the Olympiad’s structure, fields, and implementation process is provided here, while a detailed description is available in Appendix 1.

Over the past decade, the Iranian Scientific Olympiad for Medical Students has promoted inter-university collaboration, strengthened students’ scientific motivation, and supported innovative learning approaches [8]. However, studies consistently report persistent challenges, including financial and infrastructural limitations, weak planning, variable quality of training programs, student demotivation, and concerns about fairness in evaluation processes [9, 10]. Additional criticisms point to structural and design shortcomings, such as complex examination development, limited alignment with health system needs, and restricted participation, resulting in uneven and generally low engagement, particularly in universities outside major provincial centers [8, 11].

Multiple factors influence student participation. Barriers such as limited time, inadequate resources, and low intrinsic motivation can reduce engagement, whereas supportive environments and appropriate opportunities can promote it [12]. Insufficient participation not only hinders talent identification and development but also restricts the effective use of students’ scientific potential. Thus, identifying participation barriers and designing targeted interventions is essential.

Despite the growing body of literature on the Iranian Scientific Olympiad, previous studies have largely focused on evaluating implementation challenges, organizational outcomes, or specific aspects of the Olympiad [9, 11, 13]. These studies provide valuable insights but offer limited understanding of how individual, structural, and process-related factors interact to influence students’ decisions to participate. In addition, the perspectives of students who chose not to participate in the Olympiad have been largely overlooked, despite their potential to illuminate hidden or structural barriers to engagement. To address these gaps, the present qualitative study explores challenges and strategies for enhancing student participation in the Iranian Scientific Olympiad by incorporating perspectives from multiple stakeholder groups.

### Objective

This study aimed to explore individual, structural, and process-related barriers to participation in the Iranian Scientific Olympiad for Medical Students and to identify stakeholder-informed strategies to enhance engagement.

## Methods

### Study design

This study employed a qualitative descriptive design to explore challenges and strategies related to student participation in the Iranian Scientific Olympiad for Medical Students. Qualitative descriptive research provides a low-inference summary of participants’ perspectives using language that remains close to the data and is widely used in health and medical education research to inform policy and practice [14]. This approach was appropriate for the present study because it aimed to identify perceived challenges and feasible strategies across multiple stakeholder groups, rather than to interpret the existential meaning of a shared lived experience, and it aligns well with the use of semi-structured interviews and conventional content analysis [15].

### Study setting

The study was conducted within the Iranian medical sciences education system, overseen by the Ministry of Health and Medical Education, which includes universities offering programs in medicine and allied health disciplines such as nursing, public health, physiotherapy, and radiology. The Iranian Scientific Olympiad for Medical Students is a national academic initiative designed to support talented students through individual and team-based scientific competitions and is implemented through a multilevel structure involving university committees, faculty mentors, and national administrators. This multilevel organization informed the inclusion of multiple stakeholder groups in the present study. A detailed contextual description of the study setting is provided in Appendix 1.

### Participants and sampling

Participants included students, faculty members, educational administrators, and Olympiad officials with direct experience of, or formal involvement in, the Scientific Olympiad. A purposive sampling strategy with maximum variation was used to capture a broad range of perspectives across roles, academic disciplines, and institutional contexts, which is consistent with the aims of qualitative descriptive research [16].

Students were eligible if they were enrolled in a medical sciences university and had either participated in the Olympiad or were familiar with the program but had not participated. Faculty members and administrators were eligible if they had roles in Olympiad planning, implementation, mentoring, or evaluation at the university or national level. Both medical students and students from allied health disciplines were included to ensure disciplinary diversity.

To ensure the inclusion of contrasting and negative perspectives, a small number of students without prior Olympiad participation were recruited through convenience sampling, an approach considered acceptable for exploratory qualitative studies when combined with purposive strategies [16]. Sampling continued until data saturation was achieved, defined as the point at which no new categories or substantive insights emerged from additional interviews [17]. A total of 28 participants were interviewed.

### Data collection

Data were collected through semi-structured, in-depth interviews, which allowed exploration of participants’ perspectives while maintaining flexibility to probe emerging issues [18]. An interview guide was developed based on a literature review and expert consultation and covered perceived challenges, barriers and facilitators to participation, and suggested strategies for improvement. All interviews were conducted by the principal investigator in Persian, lasted approximately 20–65 min, and were held in quiet and private settings, either face-to-face or via secure online platforms. Prior to each interview, the study objectives were explained, and verbal informed consent was obtained, including permission for audio recording. Interviews were audio-recorded and transcribed verbatim, with transcripts checked against recordings for accuracy. Data collection continued until conceptual saturation was reached, when no new codes or themes emerged across stakeholder groups. For reporting purposes, selected quotations were translated into English by a bilingual researcher and verified against the original Persian transcripts to ensure credibility.

### Researcher reflexivity

The principal investigator had prior academic familiarity with the Olympiad context, which facilitated access to participants and understanding of the study setting. Recognizing that researcher background and assumptions may influence data collection and interpretation, reflexive practices were employed throughout the study, including memo-writing, regular discussions among the research team, and deliberate attention to divergent and negative cases during analysis [19, 20]. These strategies were used to enhance transparency and reduce the influence of individual bias.

### Data analysis

Data were analyzed using conventional content analysis, as described by Hsieh and Shannon. This approach is appropriate when existing theory or prior research on a phenomenon is limited and when categories are intended to emerge inductively from the data rather than being imposed a priori [15]. Data analysis involved familiarization with the transcripts, inductive coding of meaning units related to participation challenges and strategies, grouping similar codes into categories, and abstraction of overarching themes across stakeholder groups.

Data management and coding were supported using Atlas.ti software. To enhance analytic rigor, coding decisions and category development were reviewed by multiple members of the research team. Differences in interpretation were discussed and resolved through consensus, and an audit trail was maintained to document analytic decisions [15, 21]. Examples of the coding process aligned with the final analytical categories are provided in Appendix 2.

### Trustworthiness

Trustworthiness was ensured using Lincoln and Guba’s criteria [21], with credibility supported through data immersion, stakeholder triangulation, and attention to divergent cases, and dependability and confirmability enhanced by systematic documentation, reflexive memo-writing, and peer debriefing. Transferability was addressed through detailed descriptions of the study context and participants. Reporting transparency was further strengthened by adherence to the COREQ checklist [22].

## Results

In this qualitative study, semi-structured in-depth interviews were conducted with 28 participants from various medical universities across the country. Over 60% of participants were female, and approximately 43% were under 30 years of age. About one-quarter of the participants were officials from the Olympiad and outstanding talent committees, while roughly 14% were students with prior Olympiad participation (Table 1).

**Table 1:** Demographic characteristics of participants

Demographic characteristics		Frequency	Percentage
Gender	Female	17	60.7
	Male	11	39.3
Age	< 30	12	42.9
	31–40	4	14.3
	41–50	5	17.8
	> 50	7	25
Position	Faculty member	4	14.3
	Head of the University Olympiad Committee	7	25
	Head of an Olympiad field at the university	5	17.8
	Student participating in the Olympiad	4	14.3
	Student without prior Olympiad participation	8	28.5

Data analysis identified three overarching domains influencing student participation in the Iranian Scientific Olympiad for Medical Students: individual factors, structural factors, and process-related factors. Each domain comprised several interrelated subthemes reflecting barriers and facilitating strategies (Table 2).

**Table 2:** Individual, structural, and process-related factors influencing participation in the Iranian scientific Olympiad for medical students

Main domain	Subdomains
Individual factors	• Low self-esteem • Lack of personal prioritization • Lack of motivation
Structural factors	• Inadequate resources and infrastructure • Inadequate stakeholder participation • Lack of transparency • Lack of educational equity • Confined to a specific period • Lack of support programs • Deviation from the main mission
Process factors	• Multiple changes • Inappropriate scheduling • Poor communication • Incorrect selection of field officials • Poor preparation period • Lack of effective mentoring • Contentual and scientific flaws in the test • Unclear feedback and correction mechanism

### Individual factors affecting student participation

Individual factors were primarily related to students’ beliefs, priorities, and motivational drivers. Low self-confidence was frequently reported, particularly among students from smaller or less-resourced universities, who perceived themselves as disadvantaged compared to peers from larger institutions. One participant noted, *“Students at smaller universities think that their chances of success in competing with students from larger universities are low”* (P4). While a small number of participants suggested separating competitions based on university size to improve fairness, most interviewees opposed this view, emphasizing that such an approach could reduce competitiveness and limit students’ academic growth.

Another important subtheme was lack of personal prioritization, as academic workload and financial responsibilities limited students’ ability to engage in Olympiad activities. As one participant explained, *“Some students focus only on their specialized courses*,* while others need to work to earn money”* (P3). Insufficient motivation and perceived inequity in incentives were also influential. One interviewee stated, “*The benefits of participating in the Olympiad are superficial and not motivating”* (P22).

Although most participants emphasized low self-confidence, competing priorities, and insufficient motivation as key barriers to participation, a small number of interviewees expressed the view that highly motivated students would participate in the Olympiad regardless of personal confidence, incentives, or institutional conditions. However, this perspective was less frequently reported and did not reflect the dominant experiences described by the majority of participants.

### Structural factors affecting student participation

Structural factors operated at institutional and system levels and were described as major determinants of participation. Inadequate resources and infrastructure, including limited budgets, insufficient educational materials, and poor welfare facilities, were repeatedly cited. One student remarked, *“We had problems with basic issues such as transportation*,* facilities*,* and access to resources*,* which caused student dropout”* (P13).

Another prominent subtheme was inadequate stakeholder participation and coordination. Participants noted weak collaboration between Olympiad committees and other student or academic bodies, reducing institutional support. As one interviewee stated, *“Other student institutions should play a role*,* but in practice such participation is not observed”* (P6).

Lack of transparency, particularly in scoring and evaluation processes, undermined trust and discouraged repeat participation. One participant noted, *“There is no transparency regarding students’ scores and the marking process”* (P1). In addition, educational inequity—including unequal access to qualified faculty and reference materials—was frequently emphasized: *“We did not have a relevant professor to teach the references*,* and even the university could not provide some of the required materials”* (P13).

Participants also highlighted limited support programs for talented students and medalists, noting the absence of structured follow-up, mentorship, or academic integration after the Olympiad. Furthermore, several interviewees criticized deviation from the Olympiad’s core mission, describing an excessive focus on medal counts rather than long-term talent development and educational growth.

### Process factors affecting student participation

Process-related factors concerned the planning, implementation, and execution of Olympiad activities. Frequent and unpredictable changes in rules, references, and procedures were widely reported as discouraging. One participant explained, *“The many changes in the Olympiad*,* from the introduction of fields to the announcement of references*,* have caused students to be unmotivated. Changes should be gradual and minor”* (P11).

Inappropriate scheduling was another key issue, as Olympiad timelines often conflicted with academic courses and examinations. Participants emphasized the need for better coordination with educational administrators to reduce these conflicts. Although adjustments to the exam schedule have been attempted, informing professors and education administrators and applying flexible educational rules were suggested as strategies to facilitate participation.

The selection and engagement of field officials and mentors also influenced student experiences. Some participants reported limited faculty interest or expertise, negatively affecting preparation quality. Additionally, poor preparation periods and an overemphasis on content delivery rather than skill development were noted. A university official explained, *“Some professors are not interested enough to participate in the Olympiad. The university is sometimes forced to use interested professors even if they have less expertise or experience”* (P19).

Concerns were also raised regarding contentual and scientific flaws in the Olympiad examinations, including non-standardized questions, outdated references, and excessive reliance on memorization. Participants further highlighted weaknesses in the group-based component, particularly insufficient preparation for teamwork skills. To address this problem, modular online courses have been offered over the past two years. However, their effectiveness remains limited due to content-focused instruction, untrained faculty, and late access to materials. Incorporating interactive, problem-based methods—such as debates, case analyses, and practical exercises led by experienced professors—has been suggested to improve course quality.

Finally, the absence of a clear feedback and correction mechanism was identified as a recurring problem, limiting opportunities for learning and improvement across Olympiad cycles. One faculty member noted, “*Universities should gather student opinions and provide regular feedback to the ministry of health. Currently*,* universities are somewhat passive*” (P9).

While most participants emphasized frequent procedural changes, scheduling conflicts, and weaknesses in preparation and assessment processes as discouraging factors, a few interviewees reported relative satisfaction with recent procedural modifications, such as online courses and group activities. However, these accounts were limited and were often accompanied by concerns regarding inconsistent implementation and limited effectiveness.

### Strategies to enhance student engagement

Participants proposed a wide range of strategies aligned with the three identified domains. At the individual level, strategies focused on enhancing motivation through meaningful incentives, peer role models, and innovative engagement approaches. Structural strategies emphasized sustainable funding, stakeholder involvement, transparency, and long-term support for talented students. Process-related strategies highlighted the importance of clear roadmaps, early communication, improved preparation programs, higher-quality assessments, and systematic feedback mechanisms (Table 3).

**Table 3:** Strategies to enhance engagement in The Iranian Scientific Olympiad for Medical Students

Domain	Strategies
Individual	Providing meaningful material and non-material incentives, Using successful Olympiad participants as peer role models, Introducing the Olympiad through innovative and student-centered approaches, Facilitating engagement in extracurricular scientific activities, Supporting gradual skill development to enhance self-confidence
Structural	Allocating and transparently managing a dedicated budget, Involving stakeholders in policy-making, implementation, and monitoring, Ensuring transparency and fairness in evaluation and scoring, Establishing sustained support programs for talented students and medalists, Creating specialized elite student networks, Organizing inter-university collaborative scientific events, Fairly determining university quotas, Including faculty from multiple universities in committees, Organizing inter-university collaborative events, Engaging medalists in policy, research, and educational initiatives, Facilitating student participation in preparation programs, Evaluating university Olympiad committees using structural, process, and outcome indicator
Process	Developing a clear and stable Olympiad roadmap, Establishing a centralized and permanent information platform, Utilizing innovative and creative approaches to introduce the Olympiad to students, Announcing references and guidelines early, Improving communication and visibility through coordinated platforms, Defining transparent criteria for selecting field officials and mentors, Expanding faculty engagement and mentorship capacity, Improving access to updated scientific resources, Integrating analytical, problem-solving, and teamwork skills into preparation programs, Enhancing assessment quality with scenario-based questions, Establishing systematic feedback and correction mechanisms

## Discussion

This study demonstrates that medical students’ participation in the Scientific Olympiad is shaped by a dynamic interaction of individual, structural, and process-related factors. Rather than operating in isolation, these domains jointly influence students’ motivation, access, and sustained engagement, underscoring the need for multi-level interventions that extend beyond individual readiness to institutional arrangements and educational policies. This interpretation aligns with prior Iranian evidence showing that neglecting contextual and systemic dimensions can limit the Olympiad’s educational value despite its potential benefits [9].

At the individual level, low self-confidence, limited intrinsic motivation, and the perception that the Olympiad is reserved for elite students hinder participation, particularly among undergraduates and students from smaller universities [10, 23, 24]. Supportive learning environments—featuring achievable goals, constructive feedback, student involvement in program design, and mentoring—enhance intrinsic motivation, whereas reliance on extrinsic rewards has limited, short-term effects [25–27].

Structural factors further shaped participation patterns. Inequalities between large and small medical universities—particularly in access to experienced faculty, infrastructure, and educational resources—created uneven opportunities for Olympiad engagement. Such disparities, documented both in Iran and internationally, limit academic support and mentoring capacity and contribute to reduced motivation among students in resource-constrained settings [9, 10, 28–30]. Addressing these inequities requires equitable resource allocation and policies that account for institutional diversity rather than reinforcing excellence-based concentration.

Insufficient stakeholder involvement emerged as another structural barrier. Students’ perceptions that their voices were overlooked diminished motivation and ownership, consistent with evidence that stakeholder engagement enhances program effectiveness and satisfaction [11, 31]. Transparent feedback mechanisms, inclusion of student representatives in decision-making, and clearer accountability structures may therefore improve both efficiency and inclusiveness.

Concerns regarding fairness and transparency—particularly subjective evaluations and unclear scoring—also constrained participation. Similar challenges have been reported in Iranian Olympiad studies and international research on academic assessment systems, which emphasize that transparent criteria, independent evaluation, and detailed feedback are essential for credibility and engagement [9, 13, 32]. Implementing standardized judging protocols and clearer reporting may thus strengthen trust and participation.

Inequitable access to educational resources and experienced faculty, particularly in smaller universities, was another barrier. International evidence shows that reducing such structural obstacles increases participation among disadvantaged groups [33]. In Iran, modular online training courses have been introduced to address this inequality, but low quality has limited their impact. Enhancing course content and quality, decentralizing decision-making, and fairly allocating resources across universities are essential to improve educational equity.

Process-related barriers included limited preparation courses, incomplete study resources, absence of mock exams, and insufficient mentoring. Effective mentoring enhances success by identifying strengths, improving problem-solving, and boosting motivation [34]. Strategies include increasing professor awareness, providing comprehensive resources, conducting simulated exams, and establishing structured mentoring systems.

Beyond entry into the Olympiad, the lack of sustained support for medalists represented a missed opportunity. Participants noted that insufficient post-achievement investment diminishes motivation and underutilizes students’ scientific capacity, a concern echoed in both Iranian and international literature [8, 9, 25, 35]. Long-term financial support, research opportunities, and mentoring networks may help maintain engagement and translate achievement into sustained academic development.

Finally, deviation from the Olympiad’s core educational mission—marked by an excessive focus on rankings and medals—was perceived to undermine goals such as talent development, critical thinking, and scientific collaboration. Reframing success through qualitative indicators and aligned educational opportunities may better support sustainable learning and participation [9, 31].

Overall, increasing participation should not be viewed solely as a quantitative goal. Sustainable motivation, informed choice, and engagement of genuinely interested students are central to strengthening the Olympiad’s educational impact. Participants perceived that addressing individual, structural, and process-related factors in an integrated manner could foster a more equitable, motivating, and educationally meaningful Olympiad environment.

### Study limitations

Using semi-structured interviews, this study employed a qualitative descriptive approach to identify challenges and strategies for enhancing student participation, providing practical insights for managers and policymakers. Limitations include limited generalizability, as mainly motivated students participated and the findings reflect perceptions rather than causal relationships. Future research using larger, mixed-methods samples and intervention studies targeting motivational, structural, and process-related strategies could provide further evidence to improve the Scientific Olympiad.

## Conclusion

Participation in the Iranian Scientific Olympiad is shaped by interconnected individual, structural, and process factors that influence engagement and success. Strengthening participation requires a multi-level approach, including fostering intrinsic motivation, ensuring fair and transparent evaluation, equitable resource allocation, and sustainable mentoring systems. Policymakers and university administrators can enhance the effectiveness and fairness of scientific Olympiads by addressing these factors through evidence-based strategies. Future research should examine the effectiveness of targeted interventions and explore participation patterns across institutions and over time.

## Supplementary Information

Below is the link to the electronic supplementary material.

Supplementary Material 1.

Supplementary Material 2.

Supplementary Material 3.

## Data Availability

No datasets were generated or analysed during the current study.
