# Supplementary material for: Challenges and strategies to enhance participation in the Iranian medical students’ scientific Olympiad: a qualitative study
Source: BMC Res Notes. 2026 Feb 28;19:159. doi: 10.1186/s13104-026-07751-4 (PMC13059186; doi:10.1186/s13104-026-07751-4)
Supplement: Supplementary file 1 — Supplementary Material 1. [file 13104_2026_7751_MOESM1_ESM.pdf]

Interview Guide for Experts

Interview Date: ___________

Participant Position/Role: ___________

We appreciate your participation in this study. The following questions aim to explore your insights and experiences regarding the Iranian Medical Students' Scientific Olympiad.

Interview Questions:

1. Could you briefly describe your background and experience in relation to the Iranian Medical Students' Scientific Olympiad?
2. What are the main challenges of the Iranian Medical Students' Scientific Olympiad? To what extent has the program succeeded in achieving its objectives?
3. In your opinion, what factors facilitate student participation in the Olympiad?
4. What do you consider the most important barriers to student participation in the Olympiad?
5. What strategies would you suggest to strengthen student participation in the Olympiad?
6. What strategies would you recommend to improve the Iranian Medical Students' Scientific Olympiad as a program?

Notes for Interviewer:

- Encourage participants to elaborate and provide specific examples.
- Probe for details on individual, structural, and process-related factors.
- Ensure confidentiality and obtain consent before recording the interview.
- Allow participants to discuss additional points they consider relevant.
