# Supplementary material for: Challenges and strategies to enhance participation in the Iranian medical students’ scientific Olympiad: a qualitative study
Source: BMC Res Notes. 2026 Feb 28;19:159. doi: 10.1186/s13104-026-07751-4 (PMC13059186; doi:10.1186/s13104-026-07751-4)
Supplement: Supplementary file 2 — Supplementary Material 2. [file 13104_2026_7751_MOESM2_ESM.docx]

**Appendix 1. Iranian Medical Sciences Education System and the Scientific Olympiad**

**Medical Sciences Education System in Iran**

The medical education system in Iran is centrally governed by the Ministry of Health and Medical Education (MOHME), which is responsible for regulating, planning, and supervising education across all medical science disciplines and academic levels within the country’s Universities of Medical Sciences. Since the formal integration of healthcare service delivery and medical education in 1985, Iran has substantially expanded its medical education infrastructure, resulting in a nationwide network of medical universities providing basic sciences, clinical, and specialized training (1).

Universities of Medical Sciences serve as the principal institutions for medical education and encompass faculties of medicine, dentistry, pharmacy, nursing, allied health sciences, and public health. Due to the administrative affiliation of hospitals and primary healthcare centers with these universities, theoretical instruction and clinical training are delivered in an integrated manner, enabling close alignment between education and service provision (1).

To ensure educational quality and consistency, national accreditation and standardization mechanisms have been established to evaluate educational programs and institutions against predefined national and internationally aligned criteria. These quality assurance processes are primarily implemented by governmental bodies affiliated with MOHME (2). At the national level, Universities of Medical Sciences are periodically assessed and benchmarked based on indicators such as accreditation outcomes, educational quality, and research performance. Based on these evaluations, universities are classified into three categories (Type I, Type II, and Type III), with Type I universities generally representing larger institutions located in provincial capitals and equipped with more extensive educational, research, and clinical resources (3).

Within this system, full-time faculty members play a central role in sustaining educational quality. Faculty responsibilities span four main domains: education, research, clinical practice, and healthcare service delivery. Although the relative emphasis of these domains differs between clinical and non-clinical disciplines, educational and research activities constitute the core criteria for faculty performance evaluation across all fields (4).

Admission to Universities of Medical Sciences is conducted through a standardized national entrance examination (Konkur), which ensures a uniform and competitive selection process for applicants across all medical and health-related disciplines, including medicine, nursing, physiotherapy, radiology, and other allied fields. Universities do not operate independent admission procedures, and student selection is based exclusively on performance within this centralized national system.

**Scientific Olympiad of Medical Sciences Students in Iran**

The Iranian Scientific Olympiad for Medical Sciences Students was established in 2007 and has been held 17 times to date, evolving into an annual nationwide academic competition focused on enhancing scientific thinking and professional competencies among medical sciences students.

**Structure and Fields**

The Olympiad includes multiple thematic fields that reflect both disciplinary and interdisciplinary knowledge and skills. In the most recent edition (17th Olympiad), competitions were held in the following seven fields:

1. Medical Education
2. Clinical Reasoning
3. Entrepreneurship
4. Interdisciplinary Convergence of Basic Sciences
5. Health System Management
6. Medical Ethics
7. Art, Media, and Campaign Design (5).

**Participant Selection and University Quotas**

Each medical university receives a quota of participants, typically ranging from 8 to 20 students, based on institutional capacity and previous performance. Universities select their participants through internal qualifying examinations and institutional screening processes. After internal selection, students undergo university-based preparatory courses designed to strengthen their knowledge and skills prior to the national rounds (6).

**Phases of the Olympiad**

Within the structured framework of Iran’s centralized medical education system, the Scientific Olympiad of Medical Sciences Students represents a national program designed to identify, motivate, and support academically talented students across medical disciplines. The Olympiad is open to all currently enrolled students at different academic levels and across all fields of medical sciences; however, participation is restricted for PhD candidates in certain domains, and some disciplines are not eligible to compete in specific Olympiad fields (6). The Olympiad is implemented through a multi-phase process at the university and national levels, comprising both individual and group competitions (7).

Phase 1: University-level Screening

The first phase is conducted internally within each medical sciences university. Based on the quota allocated to each university, eligible students participate in local screening processes for each Olympiad field. Universities are responsible for selecting the top candidates in each field in accordance with their assigned quotas. Selected students are then formally nominated to the Ministry of Health and Medical Education to advance to the national stages of the Olympiad.

Phase 2: First National Individual Round

This phase is held at the national level and includes students nominated by universities. The competition is conducted as an electronic examination, assessing field-specific knowledge and competencies. High-performing students in this round proceed along two parallel pathways:

(1) Advancement to the second national individual round, and

(2) Formation of teams consisting of three or four members to enter the group competition.

Phase 3: First National Group Round

Teams formed in the previous phase are required to develop and submit a problem-oriented and applied project. Project topics are announced by the central Olympiad committee, and teams select their project title from the predefined list. Each Olympiad field follows a specific project format tailored to its objectives. For example, in the Health System Management field, acceptable formats may include policy briefs or review studies. Projects are evaluated according to standardized criteria defined for each field.

Phase 4: Second National Individual Round

Students achieving the highest scores in the first individual round are invited to participate in the second individual stage. This phase is conducted electronically and consists of advanced, field-specific examinations. Based on final scores, participants are awarded gold, silver, and bronze medals, as well as certificates of merit.

Phase 5: Second National Group Round

Teams selected from the first group round are invited to compete in the final group stage, which is conducted in a face-to-face format. Teams present and defend their projects before expert panels, and the top three teams in each field are selected for medal awards (7).

**Eligibility Criteria**

All students enrolled in Iranian medical sciences universities are generally eligible to participate, subject to institutional and national regulations. In some Olympiad fields, PhD and postgraduate students are prohibited from registering. These students are only allowed to register in the medical ethics, entrepreneurship, and art and media fields (8).

**Aims and Significance**

The Olympiad aims to:

- Identify and nurture high-potential students in medical sciences.
- Promote advanced scientific reasoning and professional development.
- Bridge academic knowledge with practical challenges in healthcare and health systems.
- Encourage interdisciplinary collaboration and innovation.

By integrating individual competition with team-based problem-solving, the Olympiad fosters critical thinking and collaborative skills essential for future healthcare leaders (7).

**Recent Innovations**

Recent editions have introduced flexible options such as choice of testing center independent of their universities, enhancing accessibility for participants across regions. Also, Training courses are also available to all participants in a modular and virtual format. In the field of entrepreneurship education, measures have been implemented to move away from rote learning and toward the practical training and acquisition of entrepreneurial skills. Accordingly, students are required, from the outset, to form teams, engage in idea generation, and develop their own business plans (6, 9).

**The role of medical universities in the Iranian Medical Students' Science Olympiad**

Universities of Medical Sciences play a pivotal role in the implementation of the Scientific Olympiad at the institutional level. Their responsibilities include organizing internal screening examinations, delivering preparatory educational programs, providing academic counseling and support, and nominating selected students in each Olympiad domain to MOHME. Although the mechanisms for selecting university representatives vary across institutions, commonly applied criteria include performance in written examinations, academic portfolios, and demonstrated competencies such as teamwork, research capability, and presentation skills (6).

**The role of faculty members in the Iranian Medical Students' Science Olympiad**

Following the annual announcement of Olympiad domains and the corresponding reference materials by MOHME, universities appoint experienced and qualified faculty members to coordinate each domain. These faculty coordinators are responsible for organizing instructional sessions, teaching the designated reference materials, administering mock examinations, and conducting preparatory and remedial training activities. Faculty participation in Olympiad-related activities is typically recognized through workload equivalency arrangements or financial compensation mechanisms (6).

**Iranian Medical Students' Science Olympiad Points for Medal-Winning Students**

At the national level, students who achieve top ranks in the Scientific Olympiad receive a range of supportive incentives. These include cash prizes and certificates of appreciation awarded by the MOHME Olympiad Committee, additional financial rewards, membership of top-ranked individual and team winners in the Office of Talented Students, eligibility for benefits and facilities provided by the National Elites Foundation (subject to meeting required score thresholds), facilitated pathways for progression to higher academic levels, and institutional incentives offered by Universities of Medical Sciences (6).

**References:**

1. Khanipoor F, Bazrafkan L, Aramesh S, Shojaei M, Ghasemi A. A study of medical students’ experiences at Shiraz University of medical sciences from the implementation of integration in medical education: a qualitative study. BMC Medical Education. 2024;24(1):1042.

2. Yousefy A, Changiz T, Yamani N, Zahrai R, Ehsanpour S. Developing a holistic accreditation system for medical universities of the Islamic Republic of Iran. East Mediterr Health J. 2009;15(3):650-7.

3. Sadeghi-Bazargani H, Bakhtiary F, Golestani M, Sadeghi-Bazargani Y, Jalilzadeh N, Saadati M. The research performance of Iranian medical academics: a National Analyses. BMC Medical Education. 2019;19(1):449.

4. Kumar A, Atwa H, Shehata M, Al Ansari A, Deifalla A. Faculty development programmes in medical education in the Eastern Mediterranean Region: a systematic review. Eastern Mediterranean Health Journal. 2022;28(5):362-80.

5. Secretariat of the Scientific Olympiad of Medical Sciences Students. Guidelines for the first individual stage of the 17th Science Olympiad. 2025 [cited 2025 Dec 25]. [Available from: <https://medolympiad.behdasht.gov.ir/%D8%A7%D8%AE%D8%A8%D8%A7%D8%B1/%D8%A8%D8%A7%D8%B1%DA%AF%D8%B0%D8%A7%D8%B1%DB%8C-%D8%B4%DB%8C%D9%88%D9%87-%D9%86%D8%A7%D9%85%D9%87-%D9%87%D8%A7%DB%8C-%D9%85%D8%B1%D8%AD%D9%84%D9%87-%D8%A7%D9%88%D9%84-%D8%A7%D9%86%D9%81%D8%B1%D8%A7%D8%AF%DB%8C-%D9%87%D9%81%D8%AF%D9%87%D9%85%DB%8C%D9%86-%D8%A7%D9%84%D9%85%D9%BE%DB%8C%D8%A7%D8%AF-%D8%B9%D9%84%D9%85%DB%8C>.

6. Guilan University of Medical Sciences. Introducing the Iranian Medical Students’ Scientific Olympiad. Guilan, Iran: GUMS; 2023 [cited 2025 Dec 25]. [Available from: <https://edc.gums.ac.ir/%D9%88%D8%A7%D8%AD%D8%AF-%D8%A7%D9%84%D9%85%D9%BE%DB%8C%D8%A7%D8%AF>.

7. SSecretariat of the Scientific Olympiad of Medical Sciences Students. Regulations of the Scientific Olympiad of Medical Sciences Students in Iran. Tehran, Iran: Ministry of Health and Medical Education; 2018.

8. Secretariat of the Scientific Olympiad of Medical Sciences Students. Conditions for participants of the 17th Medical Science Students Science Olympiad. Tehran, Iran: Ministry of Health and Medical Education; 2025.

9. Secretariat of the Scientific Olympiad of Medical Sciences Students. Guidelines for the field of entrepreneurship and artificial intelligence. Tehran, Iran: Ministry of Health and Medical Education; 2025.
