# Supplementary material for: Challenges and strategies to enhance participation in the Iranian medical students’ scientific Olympiad: a qualitative study
Source: BMC Res Notes. 2026 Feb 28;19:159. doi: 10.1186/s13104-026-07751-4 (PMC13059186; doi:10.1186/s13104-026-07751-4)
Supplement: Supplementary file 3 — Supplementary Material 3. [file 13104_2026_7751_MOESM3_ESM.docx]

**Appendix 2. Example of the Coding Process**

Table S2 provides an example of the coding process used in this study, illustrating how meaning units from participant interviews were transformed into initial codes, subcategories, and final categories.

Table S2. Example of the coding process used in this study, showing the transformation from meaning units to initial codes, subcategories, and final categories.

| Meaning Unit (Excerpt): Actual quotation from participant | Initial Code: Label assigned to the meaning unit | Subcategory: Grouping of similar initial codes | Final Category: Higher-level abstraction (theme) |
| --- | --- | --- | --- |
| “Sometimes I feel that the Olympiad is not for me, as it seems to be dominated by a small group of highly academic students who are far more advanced than I am.” | Low perceived self-competence | Low self-esteem | Individual factors |
| “Students pursue different goals during their studies: some focus solely on their textbooks, others prioritize work and income generation, and some are mainly concerned with completing their studies as quickly as possible to obtain a degree.” | Different student goals | Lack of personal prioritization | Individual factors |
| “In larger universities, students have greater access to professors who are academically stronger and more experienced, and this helps them become better prepared.” | Unequal institutional capacity | Lack of educational equity | Structural factors |
| “The Olympiad elevates students to a high level but then leaves them there … After winning a medal, I was highly motivated and eager to pursue more meaningful and impactful work, but there was no structured pathway to build on that momentum.” | Fragmented institutional support | Lack of support programs | Structural factors |
| “One missing element is collaboration among universities; however, a competitive atmosphere discourages this, as institutions tend to focus on showcasing themselves through winning more medals rather than working together.” | Mission drift | Deviation from the main mission | Structural factors |
| “With each change in Olympiad leadership, domains are added or removed and the guidelines are completely revised, making it impossible to plan with confidence for the following year.” | Instability in implementation | Multiple changes | Process factors |
| “Universities should make better use of students’ own experiences in Olympiad units; having gone through the process, they understand the pathway and its challenges and can provide valuable support.” | Absence of structured mentorship | Lack of effective mentoring | Process factors |
| “Student score reports should be shared transparently so participants can learn from their performance; even medal winners may not know which questions they answered incorrectly, and the lack of transparency also fuels speculation and mistrust.” | Lack of post-exam feedback | Unclear feedback and correction mechanism | Process factors |
